# Validation of the differential prognostic impact of type 1/type 1-like versus type 2/type 2-like *CALR* mutations in myelofibrosis

**DOI:** 10.1038/bcj.2015.90

**Published:** 2015-10-16

**Authors:** P Guglielmelli, G Rotunno, T Fanelli, A Pacilli, G Brogi, L Calabresi, A Pancrazzi, A M Vannucchi

**Affiliations:** 1 Dipartimento di Medicina Sperimentale e Clinica, Centro di Ricerca e I nnovazione per le Malattie Mieloproliferative, Azienda Ospedaliera Universitaria Careggi, Università degli Studi, Firenze, Italy

The discovery of mutations in calreticulin (*CALR*) in patients with primary myelofibrosis (PMF)^1, 2^ prompted a reappraisal of the clinical correlates and prognostic impact of the so-called driver mutations that include *JAK2*V617F, *MPL*W515L/K/A and *CALR* in ~60%, 5–10% and 20–25% of patients, respectively. As compared with their *JAK2*V617F counterpart, PMF patients harboring *CALR* mutations showed younger age, higher platelet and lower hemoglobin and leukocyte counts. The cumulative incidence of anemia, leukocytosis and thrombocytopenia was significantly lower in *CALR*-mutated patients who were also less likely to be red cell transfusion-dependent;^3, 4^ in addition, they had significantly longer large splenomegaly-free survival compared with the other genotypes as well as patients lacking the three driver mutations (triple-negative (TN) patients).^3, 4^ Interestingly, spliceosome mutations were significantly less represented in *CALR-*mutated patients; however, no additional molecular or cytogenetic correlate was highlighted.^3^ These data suggested a milder disease in patients harboring the *CALR* mutation, and conceivably the presence of *CALR* mutation was associated with better overall survival (OS) when compared with *JAK2*V617F- and *MPL*W515-mutated patients, and particularly TN patients.^3, 4^ In multivariable analysis, *CALR* mutations had a favorable impact on survival that was independent of International Prognostic Scoring System (IPSS), Dinamic IPSS (DIPSS)^4^ or DIPSS-plus risk stratification,^3^ and also of *ASXL1* mutation, known for its dismal impact on survival in PMF.^5^ At this regard, we found that DIPSS-plus-independent OS was significantly longer in *CALR*-mutated/*ASXL1*-unmutated compared with *CALR*-unmutated/*ASXL1*-mutated patients.^6^

Two main types of *CALR* mutations have been described until now, known as type 1 (a 52-bp deletion; p.L367fs*46) and type 2 (a 5-bp TTGTC insertion; p.K385fs*47); more than 50 indels were subsequently reported that can be grouped as type 1- or type 2-like based on their molecular characteristics. The frequency of type 1 mutation is reported to be higher in PMF (60–80%)^4, 7^ compared with essential thrombocythemia (39–61%),^4, 8, 9, 10, 11^ a part for a series including Chinese patients where no difference was noticed.^12^ Interestingly, known mutations generate a novel amino-acid C terminus of the protein with loss of the KDEL motif and replacement of negatively with positively charged or neutral amino acids, whose proportion however varies according to the type of mutation and might underlie emerging differences in the clinical correlates and prognostic impact associated with the two types of mutations. In this regard, it has been shown recently that the phenotype of mice expressing type 1 or type 2 mutation by retroviral transfer differs in that type 1 was associated with marked thrombocytosis and rapid progression to a myelofibrosis-like disease (with subsequent thrombocytopenia, splenomegaly and anemia), whereas type 2 was a milder phenotype with less thrombocytosis and reduced propensity to evolve.^13^ In a study of 617 PMF patients, of whom 140 were *CALR-*mutated, we found that patients harboring type 1 *CALR* mutation (*n*=101) had a better OS compared with those harboring *JAK2*V617F mutation (*n*=399; *P*<0.001), whereas no difference was observed between *CALR* type 1 and type 2 (*n*=22), and as well as between patients with *CALR* type 2 mutation and those with *JAK2*V617F; such differences remained also after adjustment for the DIPSS score.^4^ A significant impact of *CALR* type 1 versus type 2 was observed by Tefferi *et al.*^7^ in a comparison of 76 and 10 *CALR*-mutated patients, respectively, that also included 196 patients harboring *JAK2*V617F mutation. Survival was longer in *CALR* type 1 compared with both *CALR* type 2 (hazard ratio (HR) 2.5, 95% confidence interval (CI) 1.1–5.4) and *JAK2*V617F (HR 2.8, 95% CI 1.9–4.2); in multivariable analysis that included DIPSS and *ASXL1* mutational status, *JAK2*V617F versus *CALR* type 1 mutation, DIPSS and *ASXL1* all remained independently predictive of shortened survival. At variance with the above results, a shorter survival associated with *CALR* type 1 mutation (HR 36.3, 95% CI 4.0–324.7) was reported by Cabagnols *et al.*^11^ in a study that included 45 type 1 and 8 type 2 PMF patients. On the other hand, by comparing 98 type-1/type 1-like with 15 type 2/type 2-like *CALR*-mutated patients, Tefferi *et al.*^14^ showed that the median survival of type 1/type 1-like patients (13.7 years) was significantly better than type 2/type 2-like (3.5 years) ones, that on turn had survival superimposable to patients harboring *JAK2*V617F mutation (4 years). The difference between the two types of *CALR* mutation remained significant after adjusting for age, *ASXL1* or *EZH2* mutations.

Owing to the above conflicting results, the aim of this study was to evaluate the prognostic impact, if any, of the two different types of *CALR* mutations in a series of 396 PMF patients with a diagnosis of PMF made according to the 2008 World Health Organization criteria seen in our center. The mutational status of *CALR* was determined at diagnosis using previously described procedure of high-resolution capillary electrophoresis of fluorescent dye-labeled PCR amplicon of *CALR* exon 9;^1^ samples with abnormal peaks were subjected to conventional bidirectional Sanger sequencing. The mutated *CALR* allelic burden was estimated by peak area integration from capillary electrophoresis plot. The *JAK2*V617F mutational status was determined using real-time polymerase chain reaction, as described.^15^ Comparisons of quantitative variables between groups of patients were carried out using the nonparametric Wilcoxon rank-sum test. The cumulative probability of survival (OS) was estimated using the Kaplan–Meier method. Differences in OS between the groups were compared by using a log-rank test in univariate analysis. The SPSS package (V.22; Chicago, IL, USA) was used for statistical analysis.

Out of the entire series, we found 251 patients (63.4%) harboring *JAK2*V617F mutation, 21 (5.3%) with a *MPL*W515 mutation, 50 (12.6%) lacking any known driver mutation and 74 (18.7%) who harbored a *CALR* mutation, of whom 53 (71.6%) were type 1/type 1-like and 21 (28.4%) were type 2/type 2-like. There was no statistically significant difference between the two groups harboring *CALR* mutation as regards the various hematologic and clinical variables reported in Table 1. On the other hand, patients harboring both *CALR* mutation types differed from the *JAK2*V617F-mutated counterpart for younger age, lower leukocyte and higher platelet counts; males were more represented among Type 1/type 1-like patients than the other genotypes. The risk stratification of the patients was performed using the IPSS score at the time of diagnosis, coincident with the genotyping. There were less IPSS intermediate-2 and high-risk patients in *CALR* type 1/type 1-like mutational group (20.8%) as compared with type 2/type 2-like (38% *P*=0.04) and *JAK2*V617F mutational groups (44.2% *P*=0.193).

One hundred three patients died (26.0% of total), accounting for 17%, 47.6% and 33.5% of the patients harboring *CALR* type 1/type 1-like, *CALR* type 2/type 2-like and *JAK2*V617F mutation, respectively. The difference was statistically significant for the comparison of type 1/type 1-like versus both type 2/type 2-like (*P*=0.007) and *JAK2*V617F (*P*=0.011), unlike for type 2/type 2-like versus *JAK2*V617F mutation (*P*=0.142). Kaplan–Meier estimates of survival are shown in Figure 1. The median survival of patients harboring *CALR* type 1/type 1-like mutation was 26.4 years (range, 15.5–37.3) versus 7.4 years (4.6–10.2) for *CALR* type 2/type 2-like and 7.2 years (5.7–8.6) for *JAK2*V617F. The difference between type 1/type 1-like and the other two groups was highly significant (*P*<0.0001). The corresponding HR (95% CIs), taking the *CALR* type 1/type 1-like group as the reference, was 4.9 (95% CI, 1.8–12.9) and 6.0 (95% CI, 2.7–13.4) for *CALR* type 2/type 2-like and *JAK2*V617F-mutated patients, respectively. Survival of patients with *CALR* type 2/type 2-like mutation did not differ from *MPL*W515-mutated patients (*n*=21; median survival 14.7 years; HR 6.0, 95% CI, 2.2–16.3), whereas, as previously reported, the worst survival (median, 2.0 years; 1.6–2.4) was observed in TN patients (HR 20.6, 95% CI, 8.9–48.4). In a multivariable Cox proportional hazard regression model considering type of mutation (*CALR* type 1/1-like, *CALR* type 2/2-like and *JAK2*V617F) and the IPSS score, *CALR* type 2/type 2-like (HR 4.4, 95% CI 1.6–11.7) and *JAK2*V617F (HR 3.8, 95% CI 1.7–8.7) retained significant IPSS-independent prognostic impact on survival. Finally, there were more leukemia transformations in *CALR* type 2/type 2-like mutational group than in type 1/type 1-like (19 versus 5.7%) and *JAK2*V617F (6.8%) one; however, the low number of events warrants caution in the interpretation of the data.

In summary, our findings support previous report that the prognostic advantage of *CALR* mutation in PMF regards only patients harboring type 1/type 1-like mutation, as the survival of those harboring type 2/type 2-like mutation does not differ from *JAK2*V617F-mutated patients. However, even this study, although including the highest number of patients to date, suffers from a relatively small number of *CALR* type 2/type 2-like mutated cases, which might hamper a clean understanding of the relevance of such mutational status for survival; furthermore, the mutational landscape of PMF is highly complex because of the occurrence of several other subclonal mutations that exert a strong prognostic impact, which should be taken into an account. Therefore, larger patient series and inclusion of as many as possible mutational data in a multivariable model are needed before such information can be translated to the clinical practice to inform therapy.

## Figures and Tables

**Figure 1:**
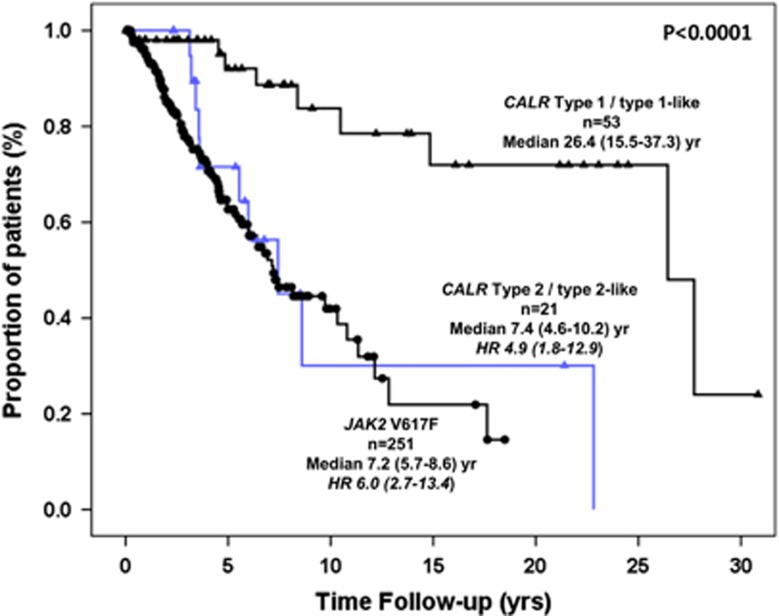
Survival data of PMF patients stratified according to their *CALR* type 1/type 1-like, *CALR* type 2/type 2-like and *JAK2*V617F mutational status.

**Table 1:** Clinical characteristics and outcome of PMF patients stratified according to their *CALR* type 1/type 1-like, *CALR* type 2/type 2-like and *JAK2*V617F mutational status

*Variables*	*CALR mutated*			*JAK2V617F mutated*	P*-value*		
	*Type 1/1-like*	*Type 2/2-like*	*P*		*CALR versus JAK2V617F*	*CALR Type 1/1-like versus JAK2V617F*	*CALR Type 2/2-like versus JAK2V617F*
*N* (% of total)	53 (13.4%)	21 (5.3%)	—	251 (63.4%)	—	—	—
Age in years; median (range)	53.3 (21–84)	61.2 (27–82)	0.210	66.7 (28–90)	**<0.0001**	**<0.0001**	**0.027**
Males; *n* (%)	25 (47.2%)	13 (61.9%)	0.253	159 (63.3%)	0.090	**0.022**	0.534
Hemoglobin, g/l; median (range)	112 (64–140)	108 (80–150)	0.958	120 (40–175)	0.637	0.362	0.704
Leukocytes, × 10^9^/l; median (range)	7.0 (1.6–18.9)	7.7 (4.2–18.1)	0.159	10.0 (1.9–96.2)	**<0.0001**	**<0.0001**	**0.047**
Platelets, × 10^9^/l; median (range)	441.5 (59–1563)	629.5 (22–1302)	0.112	308.0 (23–2011)	**<0.0001**	**0.024**	**<0.0001**
Circulating blasts ⩾1% *n* (%)	6 (12.8%)	5 (27.8%)	0.149	45 (18.7%)	0.356	0.227	0.252
Constitutional symptoms; *n* (%)	14 (26.4%)	4 (19.0%)	0.505	93 (37.1%)	0.106	0.093	0.074
							
*Cytogenetic categories;* n *(%)* 'N*' evaluable=231*							
Abnormal Unfavorable karyotype	7 (22.6%)	3 (23.1%)	0.971	45 (30.6%)	0.267	0.286	0.171
High/very high	4 (12.9%)	2 (15.4%)	0.827	16 (10.9%)	0.858	0.474	0.444
							
*IPSS* *risk group;* n *(%)*							
Low	26 (49.0%)	9 (43.0%)	0.170	58 (23.1%)	**0.001**	**<0.0001**	0.193
Intermediate-1	16 (30.2%)	4 (19.0%)		82 (32.7%)			
Intermediate-2	9 (17.0%)	4 (19.0%)		56 (22.3%)			
High	2 (3.8%)	4 (19.0%)		55 (21.9%)			
Progression to leukemia; *n* (%)	3 (5.8%)	4 (19.0%)	0.08	17 (6.8%)	0.105	0.540	0.066
Death; *n* (%)	9 (17.0%)	10 (47.6%)	**0.007**	84 (33.5%)	**0.017**	**0.011**	0.142

Abbreviation: PMF, primary myelofibrosis. Bold values denote statistically significant difference.
